# Two invasions at once: update on the introduction of the invasive species *Aedes aegypti* and *Aedes albopictus* in Cyprus – a call for action in Europe[Fn FN1]

**DOI:** 10.1051/parasite/2023043

**Published:** 2023-09-29

**Authors:** Marlen Ines Vasquez, Gregoris Notarides, Sotiris Meletiou, Eleni Patsoula, Mihaela Kavran, Antonios Michaelakis, Romeo Bellini, Toumazis Toumazi, Jeremy Bouyer, Dušan Petrić

**Affiliations:** 1 Department of Chemical Engineering, Cyprus University of Technology 3020 Limassol Cyprus; 2 Faculty of Public Health Policy, Laboratory for Surveillance of Infectious Diseases, School of Public Health, University of West Attica 11521 Athens Greece; 3 University of Novi Sad, Faculty of Agriculture, Center of Excellence One Health – Vectors and Climate 21101 Novi Sad Serbia; 4 Laboratory of Insects and Parasites of Medical Importance, Benaki Phytopathological Institute 14561 Athens Greece; 5 Centro Agricoltura Ambiente “G. Nicoli” 40014 Crevalcore Italy; 6 UMR Astre (Animals, Health, Territories, Risks, Ecosystems), Cirad, Inrae, Univ. Montpellier 34398 Montpellier France; 7 Insect Pest Control Laboratory, Joint FAO/IAEA Centre of Nuclear Techniques in Food and Agriculture, International Atomic Energy Agency A-1400 Vienna Austria

**Keywords:** Invasive mosquitoes, *Aedes aegypti*, *Aedes albopictus*, Surveillance, Eradication

## Abstract

*Aedes aegypti*, the yellow fever mosquito and *Aedes albopictus*, the tiger mosquito, continue to expand their geographical distribution, reshaping the European epidemiological risks for mosquito-borne diseases. The reintroduction of *Aedes aegypti* near the airport and port in Larnaka and the detection of *Aedes albopictus* near the marina and old port of the Limassol area in Cyprus are reported herein. The measures taken to investigate these events included (i) communication to health authorities, (ii) expert on-site visits and verification of findings, (iii) enhanced active surveillance, and (iv) development of an Emergency Action Plan followed by a Contingency Plan. These emergency action plans were developed to delimitate the infested areas and to prevent the spreading of the mosquito populations into new areas. The general principles are presented along with their rationale to serve as guidelines for other geographical regions targeting suppression/eradication with a sterile insect technique component. In parallel, this manuscript serves as a call for action at the European level to impede the further spread of these species and support the activities being undertaken in Cyprus to combat the incursions of *Aedes* invasive species.

## Abstract (fr)

*Aedes aegypti*, le moustique de la fièvre jaune, et *Aedes albopictus*, le moustique tigre, continuent à étendre leurs aires de distribution, redessinant les risques épidémiologiques des maladies transmises par les moustiques en Europe. La réintroduction d’*Aedes aegypti* près de l’aéroport et du port de Larnaka, et la détection d’*Aedes albopictus* près de la marina et du vieux port de la zone de Limassol à Chypre sont rapportées dans cette étude. Les mesures prises pour investiguer ces évènements ont inclus : (i) la communication avec les autorités sanitaires, (ii) des visites d’experts sur site et la vérification des résultats, (iii) une surveillance active augmentée et (iv) le développement d’un Plan d’Actions d’Urgence suivi d’un Plan de Contingence. Des plans d’action d’urgence ont été développés pour délimiter les zones infestées et prévenir l’expansion des populations de moustiques à d’autres zones. Les principes généraux sont présentés, en même temps que leur justification, pour guider d’autres zones géographiques visant la suppression / éradication avec une composante de technique de l’insecte stérile. En parallèle, cet article sert d’appel, au niveau européen, à prévenir la diffusion toujours plus importante de ces espèces et pour appuyer les activités à mener à Chypre pour combattre les incursions des espèces invasives d’*Aedes*.

## Introduction

*Aedes* invasive mosquito populations are increasingly expanding on the European continent. At least six invasive mosquito species (i.e., *Aedes atropalpus, Ae. japonicus, Ae. koreicus, Ae. aegypti, Ae. albopictus,* and *Ae. triseriatus*) are known to have been introduced or established in Europe [19]*.* Among these, the reintroduction of *Ae. aegypti* is of primary significance as, historically, it has been associated with dengue and yellow fever epidemics in southern Europe, where it was established until the 1950s [33]. *Aedes aegypti* is considered one of the most effective disease vectors due to its anthropophilic behaviour, biting indoors and outdoors, and requiring multiple bloodmeals during a single gonotrophic cycle [34]. Several outbreaks of yellow fever occurred in Gibraltar, Spain, France and Italy in the early 19th century [7, 34], which ceased after the discovery of the vaccine in the middle of the last century. Based on ECDC, as of March 2023, *Ae. aegypti* is known to be introduced in regions of Ukraine and the Netherlands and established in territories of Russia, Georgia, Turkey, Portugal and Egypt [21]. The re-emergence of *Ae. aegypti* in Egypt was reported in 2017, making it the most probable vector for the dengue outbreak in 2011 [2]. Moreover, it caused the dengue outbreak in Madeira in 2012, infecting at least 2,200 people [16] and was introduced in Marseille in 2018 [27]. *Aedes albopictus*, on the other hand, is still the fastest-spreading invasive mosquito species, capable of persisting in temperate climates due to its ecological plasticity [27], causing epidemics of dengue in Croatia, France, Italy, Portugal, and Spain [20] and of Chikungunya in France and Italy [4, 12]. It is widely distributed in Europe throughout countries with Mediterranean and temperate climates [27]. Potential epidemics of Zika cannot be ruled out through imported cases considering that both *Ae. aegypti* and *Ae. albopictus* are competent vectors as well. 

Cyprus was one of the few Mediterranean countries where, after the disappearance of *Ae. aegypti*, no invasive mosquito species were recorded. The high suitability of the island to *Ae. aegypti* from historical to recent years is well documented [39]. *Aedes aegypti* was reported on the island by Aziz in 1934 [5] and lastly by Foote and Cook in 1959 [24]. A very limited distribution of *Ae. aegypti* during surveys in 1959–1962 in Greece and Turkey is documented by Curtin [13]. The national health authorities of Cyprus carried out their first invasive mosquito surveillance in 2013–2015 (Marios Violaris, Medical and Public Health Services, Ministry of Health, pers. communication). This was followed by targeted surveillance at the main Limassol port in 2017–2018 [15]. In 2020, no invasive species were recorded during the harmonised pan-European surveillance network at the main points of entry [18]. Floodwater mosquitoes, such as *Ae. caspius* and *Ae. detritus* and *Culex pipiens* were primarily recorded in these areas [29]. In September 2020, island-wide surveillance was initiated under the UNDP Technical Committee on Health to respond to the West Nile virus epidemic in 2019 [17]. Again, the presence of invasive *Aedes* species was not recorded [36]. Following the ECDC communication on *Ae. aegypti* to the general public on 29 September 2022 [18] and the national media release on *Ae. albopictus*, in this manuscript, we provide information on the reintroduction of *Ae. aegypti*, the detection of *Ae. albopictus* in Cyprus, and actions in response to these events.

## Materials and methods

### Entomological surveillance and risk communication

**Figure 1:**
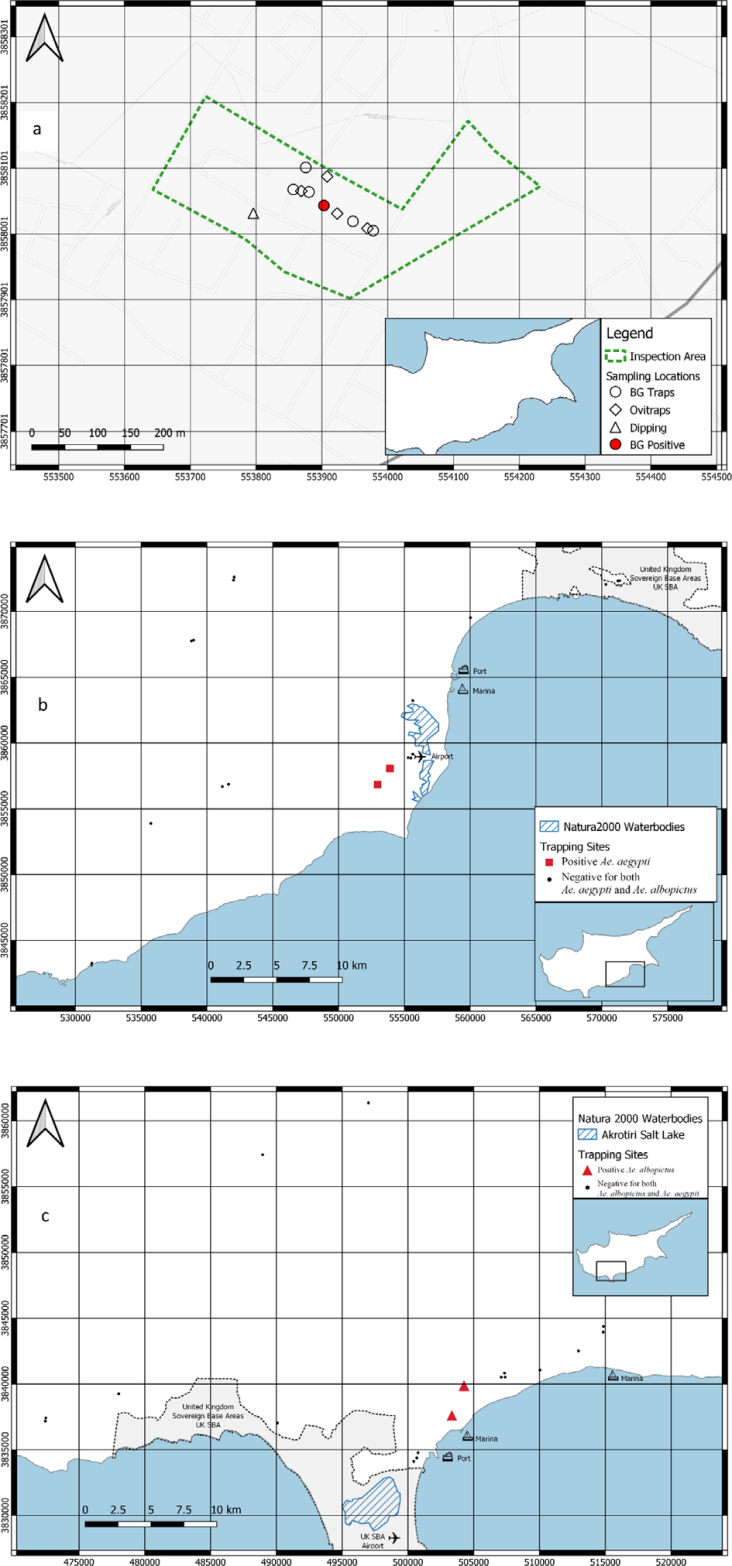
Area of Larnaka (white shapes are negative for both *Aedes aegypti* and *Ae. albopictus*, red shapes positive for *Ae. aegypti*) inspected in May 2022, followed by an enhanced active surveillance area in June and July, (a); active surveillance in Larnaka (b); and Limassol (c) in September 2022. Black dots (•) represent negative sites for both invasive species and red squares (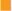) or red triangles (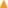) are positive sites for invasive *Aedes* *aegypti* (Larnaka) or *Aedes albopictus* (Limassol), respectively.

Following communication with the funding body and health authorities, sequential steps were taken to investigate whether *Ae. aegypti* could overwinter following the detection in late November 2021. As a first step, an expert *ad-hoc* inspection at up to a 250 m radius from the first detection point was performed in May 2022 (Fig. 1a). This involved surveillance of the drainage system and interviewing residents who were outdoors (up to ten people). Larvae were sampled using standard dippers and kept in breeders for morphological identification of L3–L4 larval instars and adults.

Moreover, Human Landing Collection (HLC) in a radius of 100 m from the first detection point was done at five sites selected by an experienced collector for 15 min/site, using an electrical aspirator. In the same area, four 1.5-L ovitraps (Isiplast, Correggio, Italy) (for seven d), six BG-sentinel traps (BGs, Biogents AG, Regensburg, Germany) and three EVS (Bioquip, Compton, CA, USA) (both for 24 h), baited with dry ice (1 kg in a container) and BG-Lure (for BG traps only) were used. As a next step, enhanced active surveillance followed in the same area with four BG (24 h) and eight ovitraps (7 days) in June and July 2022 to maximize the possibility of catching the target species.

To investigate a complaint about aggressive mosquito biting during the day in the Limassol area in May 2022, an inspection by HLC (one site for 15 min) and one BG-sentinel baited with dry ice and BG-Lure (24 h) was done near the old port and the marina, without catching specimens of invasive species. 

Active surveillance by placing BG-sentinel traps baited with dry ice (1 kg) and BG-Lure at a density of 18 km by 18 km in both Larnaka and Limassol municipalities, and the greater areas (such as municipalities of Meneou and Kiti) was performed from September 2022 (Figs. 1b and 1c). This was done in parallel with passive surveillance meaning investigations of citizen communication to local and regional health offices and information received through social networks, *e.g.*, Facebook, WhatsApp and Viber.

Communication with the local communities by health authorities and university researchers, followed by national press releases from health authorities, was done after confirmation of the overwintering for *Ae. aegypti* in the Larnaka area (September 2022) and after the presence of *Ae. albopictus* was verified in the Limassol area (October 2022)*.*

From October 2022, surveillance was performed according to a delimitation strategy for each species in their respective detection areas. The strategy includes surveillance of predefined delimitation cells (500 m × 500 m) surrounding each positive cell of up to five km distance (containment area). All adult specimens collected are transferred in a cold chain using dry ice and kept at −80 °C until identification.

### Invasive species verification

**Figure 2:**
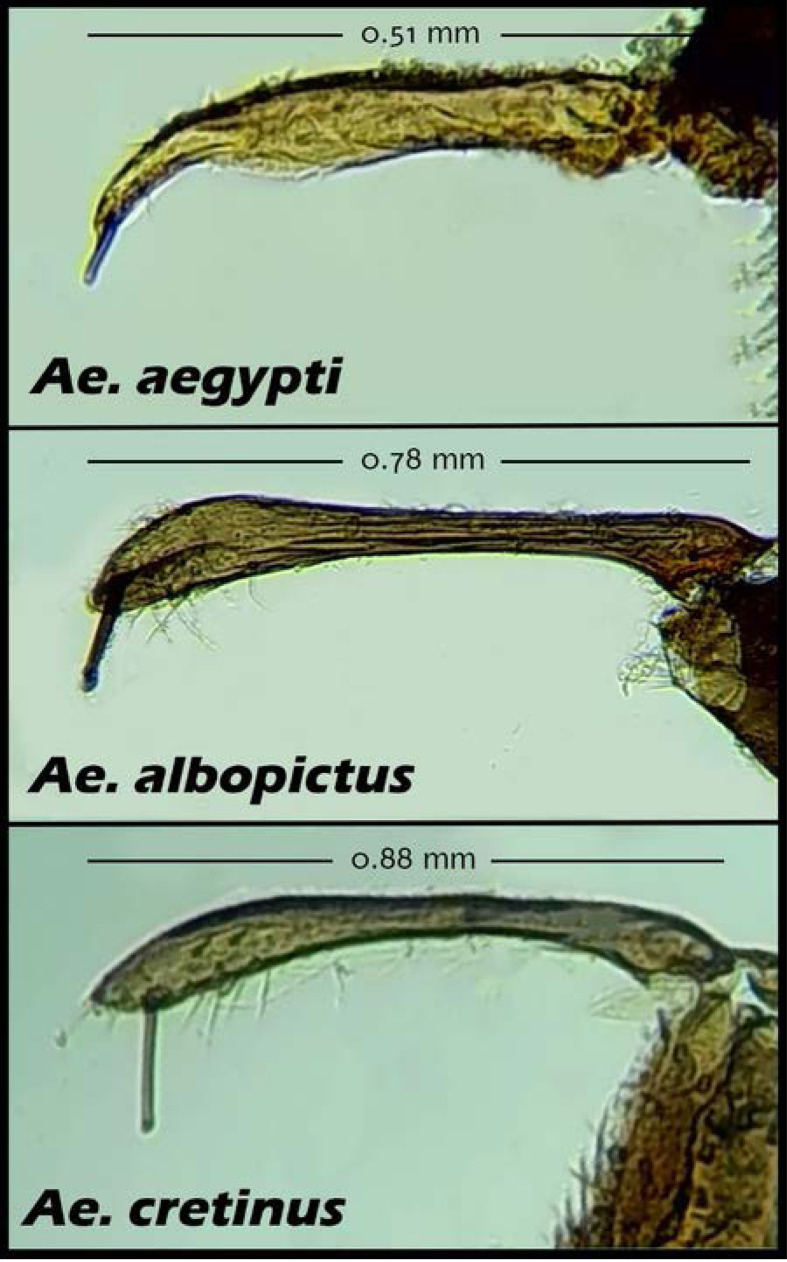
Photographs of the apical spine of the gonostylus of *Ae. cretinus*, *Ae. albopictus* and *Ae. aegypti* male genitalia. The females of *Ae. cretinus* and *Ae. albopictus* looks similar and might easily be misidentified.

Morphological identification of specimens and male genitalia (Fig. 2) was made using the key by Becker *et al.* [8] and the MosKeyTool [26]. Molecular identification was performed after DNA extraction from single adult mosquitoes using a commercial kit, following the manufacturer’s instructions (NucleoSpin Tissue, DNA Mini kit, Macherey-Nagel GmbH, Düren, Germany). A portion of the mitochondrial cytochrome oxidase I gene (COI) was amplified using primers C1-J-1718 (3′–GGAGGATTTGGAAATTGATTAGTTC–5′) and C1-N-2191 (3′–CCCGGTAAAATTAAAATATAAA-CTTC–5′) and PCR was carried out as previously described [32]. PCR products were electrophoresed, visualised and subsequently analysed by Sanger sequencing (CeMIA SA, Greece). Sequences were uploaded in GenBank under the accession numbers OP718251 for the *Ae. aegypti* cytochrome oxidase subunit isolates and OQ305997 to OQ306001 for *Ae. albopictus* cytochrome c oxidase subunit isolates.

## Results and discussion

### Entomological surveillance

The inspection in May 2022 did not confirm *Aedes* invasive species breeding sites, nor were adults collected (Fig. 1a). From a total of 353 adult specimens (225 in BG and 128 in EVS), 158 *Culex pipiens* (134 females and 24 males)*,* 12 *Culex* spp. (12 females), 165 *Aedes detritus* (165 females)*,* 12 *Aedes caspius* (12 females), six *Culiseta longiareolata* (one female and five males), 32 *Cx. pipiens* larvae, and no eggs were collected in the Larnaka area (Dromolaxia-Meneou). In June, no invasive mosquito adults or eggs were collected, but rather *Cx. pipiens* (19 females and one male), *Culex* spp. (one female) and *Ae. caspius* (nine females). On 20/7/22, a trapped female and on 27/7/22, a trapped male *Ae. aegypti* were confirmed overwintering in the Larnaka area. These specimens were collected along with *Cx. pipiens* (three females, one male), *Culex* spp*.* (one female) and *Ae. caspius* (one female). On 4/9/22, as part of the active (Fig. 1b) surveillance in the greater area, *Ae. aegypti* was trapped in the Meneou area (one female, two males) along with *Cx. pipiens* (three males), and in Kiti (one male), along with *Cx. pipiens* (one female, one male) and *Culex* spp. (one male) in Larnaka district.

A complaint from a private household in Limassol municipality in mid-September, followed by a photograph on 25/9/22 and an inspection with HLC on 26/9/22, confirmed the presence of *Ae. albopictus* (1 female sampled – Fig. 1c)*.* Another complaint and a photograph from a private household in Mesa Geitonia municipality were received on 27/9/22. On 3/10/22, the BG traps caught *Ae. albopictus* in Limassol (six males) and Mesa Geitonia (16 females and 27 males) municipalities in the Limassol district. The high number of adults indicated that the population was probably established in both municipalities.

Along with *Ae. albopictus*, *Cx. pipiens* was also trapped in Limassol (four females, two males) and Mesa Geitonia (one female).

### Invasive species verification

Depending on their condition, all specimens were morphologically identified to the genus/species level. For verification of *Aedes* invasive species, male genitalia were examined. Moreover, verification was also achieved *via* molecular identification. All samples produced visible DNA bands of expected sizes for the COI gene. Sequencing results of the COI products revealed sequences with 99.8–100% homology to the GenBank deposited sequences for *Ae. Aegypti* and *Ae. albopictus*, respectively.

The reintroduction of *Ae. aegypti,* in addition to the widespread establishment of *Ae. albopictus* in continental Europe reveals the need for a more decisive action plan to enhance proactive surveillance and rapid response systems [30]. Efforts at international ports and airports to keep these free of invasive and native mosquito vectors are a requirement under International Health Regulations [37]. Although the exact reasons for the earlier disappearance of *Ae. aegypti* from Europe is not clear, the risk of its reintroduction is high due to the increasing movement of people and goods from areas where it is present [3]. The shift of climatic conditions towards the poles facilitates, among other factors, the reintroduction of *Ae. aegypti* to new ranges, previously limited by the January 10 °C isotherm [1, 35]. As no vaccines are currently available for some of the pathogens transmitted by invasive mosquito species and conventional mosquito control methods are inefficient, integrated mosquito management, including prevention and enhanced control measures, such as active surveillance, integration of a sterile insect technique (SIT) component and a rapid response system impeding further spread, are prerequisites to prevent outbreaks [14]. This is especially pertinent for Cyprus, where the population is immunologically naïve to the diseases transferred by *Aedes* invasive mosquitos, and because the island is highly visited by tourists all year round.

### Recommendations to delimitate the incursion and prevent the spread of *Aedes* invasive species

In the absence of guidelines, and to act on the following steps to eliminate an incursion of *Ae. aegypti*, an urgent International Atomic Energy Agency (IAEA) Expert Mission under TC project CYP5020, took place in May 2022. Following WHO recommendations on developing alternative mosquito control methods [38], an Emergency Action Plan was drafted in June 2022 (four authors involved, AM, DP, RB, and JB) and adopted by the Ministry of Health in September 2022. Among the primary steps proposed were (i) to establish a Steering Committee for Vectors and Vector-borne Diseases (done in September 2022), responsible among other things for the communication strategy; (ii) to communicate to the local community using the media (performed in September 2022 in Larnaka and October 2022 in Limassol); (iii) to delimitate infested areas with a network of ovitraps and citizen science-driven HLC investigations (in progress); and (iv) to suppress the invasive species populations by door-to-door activities including breeding site removal, larviciding of permanent breeding sites with Bti and Aquatain and outdoor adulticiding with pyrethroids (in progress).

Following the verification of the presence of *Ae. albopictus* and preliminary delimitation activities, a Contingency Plan was proposed in December 2022 and adopted in April 2023 by the Ministry of Health based on the principles of area-wide integrated pest management [10, 28]. The optimal way proposed is to conduct all suppression activities through a dedicated organisational structure (incident command system), maximising efficiency. The proposed eradication strategy includes (i) an extensive public information campaign to address relevant stakeholders with emphasis on the general public; a participative approach from the community is necessary to seek their support to surveillance and the integrated control strategy; (ii) interventions on private properties; (iii) interventions on public areas; (iv) containment interventions; (v) quality control (QC) activities; and (vi) an SIT component. Intensive suppression measures are recommended to be enforced immediately in each delimitation cell when one mosquito specimen is caught in any of the four biological stages (action threshold) to try to eliminate the incursion.

#### Interventions on private properties

The interventions on private properties are recommended to be based on “door-to-door” (DtD) activities [22] that include (i) education of owners; (ii) elimination of breeding sites; (iii) larval treatment of permanent breeding sites, and (iv) adult control. Previous studies have shown that in the United States including a DtD component can suppress adult populations of *Aedes* invasive species by 25–75% [23], in Germany reduce 10× larval sites [8], and in Italy reduce 50% egg density [6]. The goal of 100% private houses covered by DtD should be sought by exploring the legal possibilities of entering facilities where citizens are unwilling to cooperate. Each DtD team should ideally have three people: one person communicating with the owner and collecting the necessary information, a second carefully inspecting the property, doing source removal and larval treatment, and a third person applying adulticide residual ultra low volume (ULV) treatment on the vegetation around the house up to a height of about 4 m from the ground, as well as on the walls, porticoes and inside the house. Since females of *Ae. aegypti* feed outdoors and indoors, the absence of residual treatment inside the homes may prevent eradication foreseen by DtD activities. Each team capacity should target around 30 objects per day (approx. 15 min/premise), and the actions should be repeated monthly in facilities of high risk where permanent breeding sites and/or vegetation are observed on the first visit. Private properties of lower risk due to no permanent breeding sites and limited vegetation must be visited every 6 months to detect any potential change detrimental to the eradication strategy.

#### Interventions in public areas

In public areas, the abandoned areas where refuse accumulates may represent a risk of holding cryptic water, which mosquitoes may exploit for larval development. It is, therefore, necessary to organise one unit equipped with a truck with a ULV generator to treat these bunches of waste with an adulticide before collecting and discharging them. Rain gutters/manholes that retain water should be treated monthly with persistent larvicides (diflubenzuron, monomolecular layers) at the maximum permitted dose.

#### Containment interventions

In addition to eradication in the target area, containment of the infestation and preventing the spread into new areas is critical to success. Knowing that vehicles can transport *Aedes* mosquitoes, their biocide treatment is recommended. This can be conducted by visiting teams from the health authorities or the inhabitants’ participation in the eradication strategy. In addition to eradication in an infested area, preventing the spread to non-infested areas and new introductions is critical to the success of an eradication programme. Treating vehicles that interact with the infested site and inspecting/treating points of entry will serve this purpose.

#### Quality control activities

Interventions should be followed by quality control (QC) performed by an independent institution (not involved in the suppression activities) responsible to the national authorities. QC of DtD activities within a week of execution in 5–10% of the riskiest facilities are needed, including surveillance of adults through HLC by aspirators (if there are no vector-borne diseases). Road drains should be checked 7–14 days after treatment by opening the drains holding water and sampling larvae/pupae with a fine mesh aquarium net from 3 to 5% of treated gutters with water. Data on all intervention activities and achieved efficacy (QC) should be kept in a dedicated database to inform and improve the eradication efforts.

#### SIT component

Eradicating invasive mosquito species with traditional methods is ineffective, as recently reviewed by Bowman *et al.* [11], because they can breed in any type of water container that is often cryptic. This might result in the failure of DtD to eliminate the target population alone, thus leading to a need to apply the SIT, which is efficient at low density and has apparent eradication properties [10]. Indicative cost efficacy of *Ae. albopictus* control in Northern Italy for traditional Integrated Mosquito Management is 1 euro per capita and achieves a 30–40% population reduction. Intensive DtD complemented with SIT costs 9–10 euros per capita, suppressing 80–90% of an insect population. After the eradication is completed, monitoring should be continued for at least three months in the initially infested areas, and if the findings are negative, the status of “*Aedes* invasive mosquito-free area” can be declared.

After declaring the status “*Aedes* invasive mosquito-free area”, preventive measures should be continued to prevent any new introduction, especially at places at higher risk of introduction that can be determined based on the origin of the collected individuals. Investigation of the origin of the collected specimens is ongoing. When finished, it might inform on the transportation means of introduction (by air or sea).

## Conclusions

The verification of the establishment of *Ae. aegypti* and *Ae. albopictus* in Cyprus initiated a data-driven eradication strategy based on area-wide integrated pest management having pillars on (i) community engagement and door-to-door activities in private and public areas; (ii) containment of the populations; (iii) QC of activities; and (iv) an SIT component. The proposed means for adequate supervision and training of the personnel involved, along with the main principles for where, when and how to apply measures for vector control, provide a tailor-made eradication scheme for Cyprus based on the current situation. Cyprus is an island, and still having the *Aedes* invasive mosquito populations limited to specific areas may present advantages in achieving its eradication goals. Successful eradication achieved in the past in continental areas [31] along with current technological developments, indicates that the process is challenging but feasible under specific environmental and geopolitical conditions and encourages the implementation of environment-safe management plans targeting suppression and possibly eradication as presented herein. At the same time, this echoes a call for action in Europe to support cooperation between countries to improve their detection capacities at points of entry and to perceive these incursions as a regional European threat. The inability to control these vectors on the islands can create “communicating vessels”, which, on the American continent (the United States, continental Latin America, and the Caribbean islands) ended the possibility of eradicating these species, maintaining a permanent reservoir for the viruses and also facilitating continental epidemics [25].
